# Supplementary material for: Molecular investigation of TSHR gene in Bangladeshi congenital hypothyroid patients
Source: PLoS One. 2023 Aug 10;18(8):e0282553. doi: 10.1371/journal.pone.0282553 (PMC10414570; doi:10.1371/journal.pone.0282553)
Supplement: Attachment — Submitted filename: Response to Reveiwer.docx [file pone.0282553.s001.docx]

**Responses to Reviewers’ Comments**

***Comment:1****. Please ensure that your manuscript meets* *PLOS ONE's style requirements, including those for file naming. The PLOS ONE style templates can be found at* 
<https://journals.plos.org/plosone/s/file?id=wjVg/PLOSOne_formatting_sample_main_body.pdf> and 
<https://journals.plos.org/plosone/s/file?id=ba62/PLOSOne_formatting_sample_title_authors_affiliations.pdf>

**Response:** Thank you for your comment. We have revised the manuscript as PLOS ONE's style.

*Comment 2. Thank you for stating the following financial disclosure: 
 "The funders had no role in study design, data collection and analysis, decision to publish, or preparation of the manuscript."*

At this time, please address the following queries:

Comment (a) Please clarify the sources of funding (financial or material support) for your study. List the grants or organizations that supported your study, including funding received from your institution.

**Response:** This study was partially funded by a grant from the University of Dhaka received from the University Grant Commission (CP-4029).

*Comment (b) State what role the funders took in the study. If the funders had no role in your study, please state: “The funders had no role in study design, data collection and analysis, decision to publish, or preparation of the manuscript.”*

**Response:** The funders had no role in study design, data collection, and analysis, the decision to publish, or preparation of the manuscript

*Comment (c) If any authors received a salary from any of your funders, please state which authors and which funders.*

**Response:** The first author was a Ph.D. student (from 2016 to 2019) under a grant from the University of Dhaka. The Ph.D. program was finished in December 2019.

*Comment (d) If you did not receive any funding for this study, please state: “The authors received no specific funding for this work.”*

**Response:** The first author received fund for genomic sequence only during PhD program during 2016-2019. No fund was available for computational study.

*Please include your amended statements within your cover letter; we will change the online submission form on your behalf.*

*3. We note that you have stated that you will provide repository information for your data at acceptance. Should your manuscript be accepted for publication, we will hold it until you provide the relevant accession numbers or DOIs necessary to access your data. If you wish to make changes to your Data Availability statement, please describe these changes in your cover letter and we will update your Data Availability statement to reflect the information you provide.*

**Response:** Thank you. We have provided all the generated and analyzed data with the revised manuscript.

*4.  PLOS requires an ORCID iD for the corresponding author in Editorial Manager on papers submitted after December 6th, 2016. Please ensure that you have an ORCID iD and that it is validated in Editorial Manager. To do this, go to ‘Update my Information’ (in the upper left-hand corner of the main menu), and click on the Fetch/Validate link next to the ORCID field. This will take you to the ORCID site and allow you to create a new iD or authenticate a pre-existing iD in Editorial Manager. Please see the following video for instructions on linking an ORCID iD to your Editorial Manager account:* [*https://www.youtube.com/watch?v=_xcclfuvtxQ*](https://www.youtube.com/watch?v=_xcclfuvtxQ)

**Response:** Thank you. Orchid iD has been inserted.

***Additional Editor Comments:***

The manuscript require substantial modifications before considering for publication.

*Comment: Please specify the region line 161, small molecule binding TM region.
The modeling of the TM region, author should consider using more than one computational tool to evaluate it, not only I-TASSER.
Since the whole work is relay on the model, the authors should have prepared the model from different tools subsequently compare and choose the most consistent one.
Table 4, write which one is acceptor and donor in proper format, the figure ligand is confusing, update it with mentioning of what are the numbers in braces. Same for table 5. It will be good to add one clear pic about the mutant position in the full length protein with more structural explanation (ref:* [*https://www.ncbi.nlm.nih.gov/books/NBK279140/*](https://www.ncbi.nlm.nih.gov/books/NBK279140/)*)
English editing is required.
Figure qualities need to be improved.*

**Response:** Thank you for your comment. The suggested corrections have been made accordingly and marked in the result section.


[Note: HTML markup is below. Please do not edit.]

**Reviewers' comments:**

Reviewer's Responses to Questions

**Comments to the Author**

*1. Is the manuscript technically sound, and do the data support the conclusions?*

The manuscript must describe a technically sound piece of scientific research with data that supports the conclusions. Experiments must have been conducted rigorously, with appropriate controls, replication, and sample sizes. The conclusions must be drawn appropriately based on the data presented.

Reviewer #1: Yes

Reviewer #2: No

Reviewer #3: Partly

**Our Response:** Yes

2. Has the statistical analysis been performed appropriately and rigorously?

 Reviewer #1: N/A

Reviewer #2: N/A

Reviewer #3: No

**Our Response:** Yes

3. Have the authors made all data underlying the findings in their manuscript fully available?

The [PLOS Data policy](http://www.plosone.org/static/policies.action#sharing) requires authors to make all data underlying the findings described in their manuscript fully available without restriction, with rare exception (please refer to the Data Availability Statement in the manuscript PDF file). The data should be provided as part of the manuscript or its supporting information, or deposited to a public repository. For example, in addition to summary statistics, the data points behind means, medians and variance measures should be available. If there are restrictions on publicly sharing data—e.g. participant privacy or use of data from a third party—those must be specified.

Reviewer #1: Yes

Reviewer #2: Yes

Reviewer #3: Yes

**Our Response:** Yes

4. Is the manuscript presented in an intelligible fashion and written in standard English?

PLOS ONE does not copyedit accepted manuscripts, so the language in submitted articles must be clear, correct, and unambiguous. Any typographical or grammatical errors should be corrected at revision, so please note any specific errors here.

 Reviewer #1: No

Reviewer #2: No

Reviewer #3: Yes

**Our Response:** Yes

5. Review Comments to the Author

Please use the space provided to explain your answers to the questions above. You may also include additional comments for the author, including concerns about dual publication, research ethics, or publication ethics. (Please upload your review as an attachment if it exceeds 20,000 characters)

***Reviewer #1: Comment:*** *Line 68-69 needs a little bit of reconstruction. Line 78, exons should be in small letters. In line no. 81, the sentence starts with an And. Line 82, what does normally placed thyroid gland mean? Line 85, how can two molecules at the same time have the highest potency? Line 139 is a bit confusing. line 150 Magnesium chloride 's formula is wrong.*

**Response:** Thank you for your comment. The suggested corrections have been made accordingly in the revised manuscript.

***Reviewer #2: Comment:*** *Overall, the study has several limitations and shortcomings that raise questions about its originality, accuracy, and clinical relevance.*
 ***Lack of originality:*** *The docking analysis has already been done by another study "DOI: 10.1089/thy.2014.0119", which raises questions about the originality and novelty of this study. Additionally, the identification of the mutations and their location in the protein have already been reported in the ClinVar database "NM_000369.5(TSHR):c.1523C>T (p.Ser508Leu), further reducing the novelty of this study.*

**Response:** Thank you for your comment. In Bangladesh, there was no genetic data are available on congenital hypothyroid patients with thyroid gland dysfunction. The present study aimed to investigate the mutation in TSHR gene and wanted to see the effect of mutation in possible drug binding site at the TSHR protein. We selected the drug and protein structure based on the above-mentioned article. However, investigating the mutational effect was unique in our study. We just investigated the mutation in Bangladeshi patients and wanted to see the effect of mutations on the drug-binding region in the TSHR protein. To do so, we have analyzed the binding affinity and interactions by molecular docking and structural and energy profiles of the protein-ligand complexes by molecular dynamics simulations followed by principle component analysis. Our intention was to explore the structure-function relationship of drugs within proteins by applying computational biology approaches. This study is important as mutation spectrum analysis helps to find out which drug is more suitable for treatment as well as to design a new drug for Congenital hypothyroidism.

***incorrect information:*** *The authors have incorrectly identified the location of one of the mutations (Asp727Glu) in the transmembrane region of the protein, when in fact it is located in the cytoplasmic region. This suggests a lack of attention to detail and accuracy in the study*.

**Response:** Thank you for your comment. The suggested corrections have been made accordingly.

***Comment:*** *Small sample size: The study only includes 21 patients with dysgenesis, which is a relatively small sample size. This may limit the generalizability and statistical power of the findings.*

**Response:** We agree with you. the small sample size is our study limitation. However, the study was cross-sectional, we recruited patients from BSMMU for over 3 years and collected specimens from 2016 to 2019. The frequency of Congenital Hypothyroidism (CH) is 1 in 3000-4000 children worldwide and we enrolled a total of 65 participants (aged less than 18 years) which represents a total of 227,500 children in Bangladesh. Among them, 21 were found as thyroid dysgenesis and also subjected to investigation of mutation in the TSHR gene. The visits were performed twice a week. In the OPD (Outpatient Department) of Pediatrics Endocrinology and NINMAS, about 50-60 children visited per day with different endocrine disorders. Among them, 1 or 2 children were confirmed cases of Congenital Hypothyroidism who visited BSMMU for their follow-up examinations. Almost all the patients were late-diagnosed and kept under treatment for the Levothyroxine (LT4) drug. If we wanted to collect more samples for CH, then we had to visit more times and enrolled more populations, which was actually not possible in this study period.

***Reviewer #3: Comment:*** *The manuscript “Molecular Investigation of TSHR gene in Bangladeshi Congenital Hypothyroid patients” reports two mutations in the transmembrane region of TSHR gene in 21 patients. The transmembrane region of TSHR proteins (wild type and mutant) are modeled using I-TASSER. The authors did docking and molecular dynamic simulations for the two small molecule drugs with the wild type as well as mutants to understand the effect of mutations. The authors used autodock vina for docking and YASARA suits for the MD simulations.
Overall application of different computational and modeling tools looks logical for the subject of the manuscript, the obtained results lack proper scientific analysis.*

*The work is clearly presented, however I have several significant questions. The crude values are mentioned in the text (Table 3) rather than the necessary information about the target (TPO protein) and template (TSHR protein) alignment. As a reader, I am unable to understand the quality of the predicted model based on the information provided.*

**Response:** Thank you for your comment. The alignment information has been added in the method section. The details about the accuracy and quality of the predicted model have been provided also.

*Comment: The full-length protein is essential to understand the function. Why is the full length of TSHR protein not modeled?*

**Response:** Thank you for your comment. Initially, the full-length protein was not modeled due to a lack of full-length experimental structure. However, now the structure is modeled using AlphaFold and superimposed the transmembrane region and found similar results.

***Comment****: The alignment information should be added and discussed. How many models were predicted by the* *I-TASSER tool. At least the user should discuss the top three models and explain the differences or similarities.*

**Response:** Thank you for your comment. Yes, we have explained the top model from the given five models for each protein, we excluded the rest due to low quality. We have explained in detail the accuracy and quality of the predicted models and marked them for your consideration.

***Comment****: Where all the models used to be built by the same template. What is the quality of the template structure? It is easy to get automated protein structure using multiple tools. If the target template alignment is low, it will be interesting to compare the models built by another popular tool to see the difference in the prediction.*

**Response:** Thank you for your comment. We have compared the I-TASSER predicted structure with AlphaFold predicted TSHR model protein.

***Comment:*** *When author discusses the C-score, it will be helpful to the reader to explain what the score means.Also, whether the small molecules were built or download from the database. The reference or web link for PyRx software can be added. There is no information about the active site nor about how many docking clusters for each of the small molecules with the protein. Are there any other docking poses found if the observed author can discuss those. Adequacy of modeling and docking were not also evaluated that is specially important for modeled proteins.*

Response: Thank you for your comment. The suggested corrections have been made accordingly and marked in the result section.

6. PLOS authors have the option to publish the peer review history of their article ([what does this mean?](https://journals.plos.org/plosone/s/editorial-and-peer-review-process#loc-peer-review-history)). If published, this will include your full peer review and any attached files.

If you choose “no”, your identity will remain anonymous but your review may still be made public.

**Do you want your identity to be public for this peer review?** For information about this choice, including consent withdrawal, please see our [Privacy Policy](https://www.plos.org/privacy-policy).

**Response:** Yes
