# Supplementary material for: Molecular investigation of TSHR gene in Bangladeshi congenital hypothyroid patients
Source: PLoS One. 2023 Aug 10;18(8):e0282553. doi: 10.1371/journal.pone.0282553 (PMC10414570; doi:10.1371/journal.pone.0282553)
Supplement: Attachment — Submitted filename: Response to Reveiwer.docx [file pone.0282553.s002.docx]

**Responses to Reviewers’ Comments**

*Additional Editor Comments:

The authors should pay more attention about the revised copy of the manuscript, the tracked word file is difficult to read and manage. Please make a clean copy word file highlighting the modifications.
Various part of the manuscript remains hard to read and lack of connection.
The authors mentioned about the addition of figures, but the figure remains same. Please update it accordingly.*

**Response:** Thank you for your comment. The suggested corrections have been made accordingly

[Note: HTML markup is below. Please do not edit.]

***Reviewers' comments:***
 *Reviewer's Responses to Questions*

***Comments to the Author***

*1. If the authors have adequately addressed your comments raised in a previous round of review and you feel that this manuscript is now acceptable for publication, you may indicate that here to bypass the “Comments to the Author” section, enter your conflict of interest statement in the “Confidential to Editor” section, and submit your "Accept" recommendation.*

*Reviewer #3: (No Response)*

**Our Response:** Yes

*2. Is the manuscript technically sound, and do the data support the conclusions?

The manuscript must describe a technically sound piece of scientific research with data that supports the conclusions. Experiments must have been conducted rigorously, with appropriate controls, replication, and sample sizes. The conclusions must be drawn appropriately based on the data presented.*

*Reviewer #3: Partly*

**Our Response:** Yes

*3. Has the statistical analysis been performed appropriately and rigorously?*

*Reviewer #3: I Don't Know*

**Our Response:** Yes

*4. Have the authors made all data underlying the findings in their manuscript fully available?

The* [*PLOS Data policy*](http://www.plosone.org/static/policies.action#sharing) *requires authors to make all data underlying the findings described in their manuscript fully available without restriction, with rare exception (please refer to the Data Availability Statement in the manuscript PDF file). The data should be provided as part of the manuscript or its supporting information, or deposited to a public repository. For example, in addition to summary statistics, the data points behind means, medians and variance measures should be available. If there are restrictions on publicly sharing data—e.g. participant privacy or use of data from a third party—those must be specified.*

*Reviewer #3: No*

**Our Response:** Yes. Additionally, we have shared the predicted proteins and docking models in the public repository, <https://figshare.com/>.

Here is the link: <https://figshare.com/s/b10e48d2a8e110cca57e>

*5. Is the manuscript presented in an intelligible fashion and written in standard English?

PLOS ONE does not copyedit accepted manuscripts, so the language in submitted articles must be clear, correct, and unambiguous. Any typographical or grammatical errors should be corrected at revision, so please note any specific errors here.*

*Reviewer #3: No*

**Our Response:** Yes

*6. Review Comments to the Author

Please use the space provided to explain your answers to the questions above. You may also include additional comments for the author, including concerns about dual publication, research ethics, or publication ethics. (Please upload your review as an attachment if it exceeds 20,000 characters)*

*Reviewer #3:* ***Comment:*** *The writing part should be improved. Please improve the clarity and explain the necessary information in detail so that the paper explains independently. As a reader I am not convinced the author's writings are clear yet.*

*What was the target template alignment percentage? Is it 30% or 70%? If the target template is very low, authors can submit* *distance guided protein structure [https://zhanggroup.org/D-I-TASSER/](https://zhanggroup.org/D-I-TASSER/). Nowadays, getting automated protein models in a few hours. Again, the authors didn’t mention the* *target template hence I cannot decide.
Additional figures would have explained the target template alignment. e.g. Like the I-TASSER help page suggests* [*https://zhanggroup.org/I-TASSER/example/*](https://zhanggroup.org/I-TASSER/example/) *. The authors can add the figure to show the target template alignment (Page 47 for the section Prediction of 3D structure of TSHR protein and ligand selection).*

**Response:** Thank you for your comment. We have substantially improved the writing. Target template alignments were above 60% (62%, 64%, and 63%) so we did not obtain structures from distance guided protein structure <https://zhanggroup.org/D-I-TASSER/> . We have clearly explained the target template alignments in section 2.4. and 3.2. and additional figures have been shown for the target template alignments.

***Comment:*** *As pointed out by the other reviewer the author’s lack attention to detail. For instance in the revised manuscript the reference 8 points out to the I-Tasser reference instead of the previous high throughput study. (Introduction, Page 44, line 111).*

**Response:** Thank you for your comment. The suggested corrections have been made accordingly in the revised manuscript.

***Comment:*** *The author’s may s**ubmit* *the predicted protein and docking models in a public repository like* [*https://figshare.com/*](https://figshare.com/) *that might be useful to other researchers*

**Response:** Thank you for your comment. We have submitted the predicted proteins and docking models in a public repository <https://figshare.com/>

Here is the link: <https://figshare.com/s/b10e48d2a8e110cca57e>

*7. PLOS authors have the option to publish the peer review history of their article (*[*what does this mean?*](https://journals.plos.org/plosone/s/editorial-and-peer-review-process#loc-peer-review-history)*). If published, this will include your full peer review and any attached files.*

**Response:** Yes
